# Interspecific Potato Breeding Lines Display Differential Colonization Patterns and Induced Defense Responses after *Ralstonia solanacearum* Infection

**DOI:** 10.3389/fpls.2017.01424

**Published:** 2017-08-28

**Authors:** Virginia Ferreira, María J. Pianzzola, Francisco L. Vilaró, Guillermo A. Galván, María L. Tondo, María V. Rodriguez, Elena G. Orellano, Marc Valls, María I. Siri

**Affiliations:** ^1^Departamento de Biociencias, Facultad de Química, Universidad de la República Montevideo, Uruguay; ^2^Unidad de Horticultura, INIA Las Brujas Canelones, Uruguay; ^3^Departamento de Producción Vegetal, Centro Regional Sur, Facultad de Agronomía, Universidad de la República Canelones, Uruguay; ^4^Instituto de Biología Molecular y Celular de Rosario (CONICET-UNR) Rosario, Argentina; ^5^Área Biología Molecular, Facultad de Ciencias Bioquímicas y Farmacéuticas, Universidad Nacional de Rosario Rosario, Argentina; ^6^Área Biología Vegetal (CONICET), Facultad de Ciencias Bioquímicas y Farmacéuticas, Universidad Nacional de Rosario Rosario, Argentina; ^7^Center for Research in Agricultural Genomics, CSIC, IRTA, UAB, UB Barcelona, Spain; ^8^Department of Genetics, Universitat de Barcelona Barcelona, Spain

**Keywords:** bacterial wilt, *Ralstonia solanacearum*, potato, *Solanum commersonii*, plant breeding, disease resistance, latent infections

## Abstract

Potato (*Solanum tuberosum* L.) is one of the main hosts of *Ralstonia solanacearum*, the causative agent of bacterial wilt. This plant pathogen bacteria produce asymptomatic latent infections that promote its global spread, hindering disease control. A potato breeding program is conducted in Uruguay based on the introgression of resistance from the wild native species *S. commersonii* Dun. Currently, several backcrosses were generated exploiting the high genetic variability of this wild species resulting in advanced interspecific breeding lines with different levels of bacterial wilt resistance. The overall aim of this work was to characterize the interaction of the improved potato germplasm with *R. solanacearum*. Potato clones with different responses to *R. solanacearum* were selected, and colonization, dissemination and multiplication patterns after infection were evaluated. A *R. solanacearum* strain belonging to the phylotype IIB-sequevar 1, with high aggressiveness on potato was genetically modified to constitutively generate fluorescence and luminescence from either the green fluorescence protein gene or *lux* operon. These reporter strains were used to allow a direct and precise visualization of fluorescent and luminescent cells in plant tissues by confocal microscopy and luminometry. Based on wilting scoring and detection of latent infections, the selected clones were classified as susceptible or tolerant, while no immune-like resistance response was identified. Typical wilting symptoms in susceptible plants were correlated with high concentrations of bacteria in roots and along the stems. Tolerant clones showed a colonization pattern restricted to roots and a limited number of xylem vessels only in the stem base. Results indicate that resistance in potato is achieved through restriction of bacterial invasion and multiplication inside plant tissues, particularly in stems. Tolerant plants were also characterized by induction of anatomical and biochemical changes after *R. solanacearum* infection, including hyperplasic activity of conductor tissue, tylose production, callose and lignin deposition, and accumulation of reactive oxygen species. This study highlights the potential of the identified tolerant interspecific potato clones as valuable genetic resources for potato-breeding programs and leads to a better understanding of resistance against *R. solanacearum* in potato.

## Introduction

Potato (*Solanum tuberosum* L.) is the third most important food crop after rice and wheat. Potato is a staple food for more than a billion people worldwide, and the global production of this crop is more than 300 million metric tons (FAOSTAT, 2014). The origin of commercial potato cultivars is limited to a restricted number of potato clones introduced from South America into Europe in the 16th century, leading to a narrow genetic base and a limited resistance to pathogens (Hooker, 1981).

Among the bacterial potato diseases, bacterial wilt caused by *Ralstonia solanacearum* ranks the first. The disease affects more than 1.5 million hectares of potato crops worldwide having a significant economic impact estimated atin $ 950 million per annum (Elphinstone, 2005). *R. solanacearum* is within the top 10 plant pathogenic bacteria because of its lethality, persistence in the environment, wide host range and broad geographic distribution (Elphinstone, 2005; Mansfield et al., 2012). This soil-borne vascular pathogen affects more than 250 monocot and dicot species in tropical, subtropical and temperate regions (Peeters et al., 2013). The bacterium infects the roots of host plants, rapidly colonizes the vascular system and releases large amounts of exopolysaccharide that prevent water flow within xylem vessels, causing wilting symptoms and subsequent plant death (Genin and Denny, 2012). *R. solanacearum* can persist, spread, and survive in different natural habitats including soil, water, and plant tissues. These outstanding multifaceted characteristics mirror the extraordinary genetic and phenotypic diversity of this xylem-invader, making difficult to achieve a sustainable disease control (Lebeau et al., 2011).

*Ralstonia solanacearum* is a species complex composed by a diverse group of strains classified in four phylotypes based on their phylogeography. Each phylotype is further subdivided in sequevars defined as groups of isolates with highly conserved DNA sequences (Fegan and Prior, 2005). A recent taxonomic revision has led to the distinction of three separate species within the species complex (Safni et al., 2014). In this new classification, the species *R. solanacearum* includes only strains from phylotype II with origin in South America. The novel species *R. pseudosolanacearum* was defined to include strains from phylotypes I and III, and strains from phylotype IV were assigned to the species *R. syzygii* (Safni et al., 2014). In Uruguay, as well as in other cold and temperate regions of the world, potato crops are mainly affected by *R. solanacearum* strains from the phylotype IIB, sequevar 1 (Siri et al., 2011).

The most economical, environmentally friendly, and effective way to control bacterial wilt in various crops relies in the use of cultivars with resistance (Boshou, 2005; Huet, 2014). Wild *Solanum* species and primitive forms of cultivated potato are considered an invaluable and diverse source of genetic variation for potato breeding for resistance to different pests and diseases (Machida-Hirano, 2015). Potato stands out among all other crops considering the genetic diversity and potential of available germplasm for breeding purposes. Bacterial wilt resistance sources have been identified in several tuber-bearing cultivated and wild *Solanum* species including *S. phureja*, *S. stenotomum*, *S. acaule*, *S. bulbocastanum*, *S. clarum*, *S. chacoense*, and *S. commersonii* (Machida-Hirano, 2015). However, the resistance from these sources was variable depending on pathogen strain and environmental conditions, making breeding potato for bacterial wilt resistance challenging (Patil et al., 2012). Although some potato varieties with moderate to highly levels of bacterial wilt resistance were released, dragging undesirable agronomic traits together with the occurrence of latent infections in tubers are still major problems (Huet, 2014). Progress obtained so far shows that bacterial wilt resistance available in *Solanum* wild species has not been fully exploited, suggesting that diversifying the genetic basis for both disease resistance and agronomical traits is a challenge for potato breeding programs.

*Solanum commersonii* Dun is a tuber-bearing wild species with high potential as bacterial wilt resistance source for potato breeding. This species is widely distributed and adapted to our environmental conditions and harbors many desirable traits, including cold tolerance, and resistance to several diseases including bacterial wilt (Laferriere et al., 1999; Carputo et al., 2009; Siri et al., 2009). Introgression of resistance through the potato breeding program in Uruguay makes use of the high genetic diversity available in this wild species (Pianzzola et al., 2005; Siri et al., 2009). The breeding scheme involves conventional interspecific crosses exploiting the occurrence of non-reduced gametes and using *S. phureja* as a bridge species, to overcome crossing barriers between *S. commersonii* and the cultivated potato. Selected F1 hybrids from *S. commersonii* × *S. phureja* progenies were backcrossed with the cultivated potato to obtain the so-called BC1 and recurrent backcross generations were obtained after crossing BC1 plants with *S. tuberosum* genotypes (Gaiero et al., 2017). These backcrosses have resulted in advanced interspecific clones with high bacterial wilt resistance and low frequency of latent infections (unpublished data).

Knowledge on pathogen distribution and multiplication in plant tissues is critical to fully exploit potential of sources of bacterial wilt resistance through breeding programs. Bacterial wilt disease progress was previously described in susceptible and resistant tomato genotypes suggesting that resistance in this crop is related with limitation of bacterial spread in the stems (Grimault and Prior, 1993; Grimault et al., 1994a; Nakaho et al., 2004). Physical barriers including tyloses production and cell wall reinforcement were found to play important functions in preventing *R. solanacearum* dissemination in vascular tissues (Grimault et al., 1994b; Nakaho et al., 2000). In a recent study, bacterial wilt resistance in tomato plants was not attributed to a limited bacterial movement in the stems but to restriction of root colonization by the pathogen (Caldwell et al., 2017). In resistant tomato cultivars, a delay in colonization of the root vascular cylinder was found, and once bacteria enter the root vascular tissue, colonization in the vasculature was spatially restricted (Caldwell et al., 2017).

In contrast, little knowledge is available regarding defense responses in potato, and the infection process in resistant sources is not well understood. Recently, transcriptomics studies have been conducted in resistant *S. commersonii* genotypes, allowing the identification of hundreds of candidate genes proposed to be involved in resistance to bacterial wilt in this wild species (Narancio et al., 2013; Zuluaga et al., 2015). Previously we developed a new screening approach to follow pathogen colonization in potato germplasm by live imaging using a luminescent *R. solanacearum* reporter strain (Cruz et al., 2014). This method allows the detection of latent infections in roots and stems tissues of asymptomatic tolerant plants and was proposed as an efficient tool for resistance screenings in potato breeding programs (Cruz et al., 2014). Here, we extend this approach to evaluate in detail the *R. solanacearum* colonization, dissemination and multiplication pattern in selected potato clones with contrasting levels of bacterial wilt resistance. In addition, we used an additional reporter strain that generate fluorescence from a synthetic green fluorescence protein (GFP) gene integrated in the bacterial chromosome. Both reporter strains were used for direct and precise visualization of fluorescent and luminescent cells in plant tissues by confocal microscopy and luminometry. To gain a better understanding of this host–pathogen interaction, induced plant defenses responses were also evaluated, including callose and lignin deposition and reactive oxygen species production.

## Materials and Methods

### Bacterial Strains and Growth Conditions

*Ralstonia solanacearum* reporter strains UY031 Pps-lux and UY031 Pps-GFP were constructed and validated previously by our group (Cruz et al., 2014). The reporter systems (*LuxCDABE* operon and GFP) were introduced in a neutral genome region of *R. solanacearum* UY031, a phylotype IIB- sequevar 1 strain isolated from potato crops in Uruguay, that shows high levels of aggressiveness (Siri et al., 2011; Guarischi-Sousa et al., 2016). Reporter strains and UY031 were grown on triphenyltetrazolium chloride medium (Kelman, 1954) and incubated at 28°C for 48–72 h. Gentamicin was used for selection of reporter strains (5 and 75 μg⋅ml^-1^ in liquid and solid cultures, respectively). Optical density was measured spectrophotometrically at 600 nm to adjust bacterial suspensions for inoculation (OD_600_ of 0.1 corresponds to 10^8^ cfu⋅ml^-1^).

### Plant Materials and Growth Conditions

Four interspecific potato clones (13001.79, 13001.107, 11201.27, and 09509.6) derived from different breeding lines were selected from the National Institute for Agricultural Research (INIA, Uruguay) germplasm collection. Introgression of resistance to *R. solanacearum* from diverse wild *S. commersonii* accessions was achieved using *S. phureja* as a bridge species followed by successive backcrosses to *S. tuberosum* (Gaiero et al., 2017). The potato cultivar *S. tuberosum* cv. Chieftain was used as a susceptible control. Plants were micro-propagated *in vitro* from a node in Murashige and Skoog (MS) medium with sucrose 30 g/l and kept at 22°C with cycles of 16 h light/8 h darkness. After 3 weeks plants were sown in plastic boxes with soil mix (Tref Substrates BV, Moerdijk, Netherlands) and grown for 1 week in a greenhouse under natural light. Then, plants were placed in a growth chamber at 24°C and 65% relative humidity with a photoperiod of 16 h light/8 h darkness for one additional week prior to inoculation assays.

### Plant Inoculation

Bacterial suspensions were prepared from overnight liquid cultures of *R. solanacearum* wild type UY031 and reporter strains incubated at 28°C, and spectrophotometrically adjusted to a concentration of 10^7^ cfu⋅ml^-1^.

For bacterial wilt resistance evaluation, potato clones grown in 88-well seedbeds were soil inoculated using 1 ml of bacterial suspension of strain UY031 to reach a final density of 10^6^ cfu⋅g^-1^ (Siri et al., 2011). Plants inoculated with saline solution were considered as the negative control treatment. Two replicate trays containing eight plants each were inoculated for each clone using a completely randomized design and the experiment was performed twice. Disease progression was registered regularly until 28 days after inoculation using a scale ranging from 0 (asymptomatic plant) to 4 (all leaves wilted). The resistance level was calculated by the area under disease progress curve (AUDPC) based on the average wilt scoring for each clone. To determine the occurrence of latent infections 2-cm stem sections from asymptomatic plants were ground, streaked onto mSMSA plates (Elphinstone et al., 1996) and incubated at 28°C for 5–7 days. Asymptomatic plants were recorded as latently infected when typical *R. solanacearum* colonies were detected on the mSMSA plates.

To follow infection process, potato clones were inoculated with *R. solanacearum* reporter strains UY031 Pps-lux or UY031 Pps-GFP. Plants grown in individual pots were soil inoculated by drenching 40 ml of the bacterial suspensions into each pot to reach a final density of 10^6^ cfu⋅g^-1^. Roots were wounded before inoculation as described by Cruz et al. (2014).

For evaluation of histological effects caused by *R. solanacearum* infection, the susceptible potato cultivar Chieftain and the tolerant clone 09509.6 were soil or stem inoculated using wild type strain UY031. For stem inoculation assays, a drop (10 μl) of the bacterial suspension (10^7^ cfu⋅ml^-1^) was placed at the petiole of the third expanded leaf counting from the top of the plant, and then wounded with a needle to favor bacterial penetration. Soil inoculation was performed as described above using plants grown in individual pots. All experiments were performed using three to five replicate plants for each genotype.

All inoculation assays were performed in a growth chamber at 28°C with 65% relative humidity and a photoperiod of 16 h light: 8 h darkness.

### Bacterial Visualization and Quantification *In Planta*

For luminescence detection, plants from clones 13001.79, 13001.107, 11201.27 and Chieftain were soil inoculated using strain UY031 Pps-lux as described above. Two independent experiments were performed using six replicate plants of each clone arranged in a complete randomized design. After inoculation, bacterial cells were detected daily in plant tissues for 6 days after inoculation using the Fuji Film LAS4000 light imager system, using the same setting conditions as described by Cruz et al. (2014). In addition, luminescence was quantified in a luminometer (Berthold FB 12) from roots and 2-cm stem segments from the basal and the aerial part of the plants. Luminescence readings were expressed as RLU per milligram of fresh tissue (Cruz et al., 2014).

For fluorescence detection, plants from clones 13001.79, 13001.107, 11201.27, 09509.6 and Chieftain were soil inoculated with the reporter strain UY031 Pps-GFP and bacteria was detected in root and stems tissues 2 and 7 days post inoculation. Several experiments with different combinations of clones were performed using three to five replicate plants for each combination of clone/time arranged in a complete randomized design. Each clone was assessed in at least two independent experiments. Stems and roots were weighed and washed with tap water, disinfected with sodium hypochlorite 1% for 1 min, washed again and dried with sterile absorbent paper. Using a previously disinfected scalpel, 2-cm stem segments were cut from 1 cm above ground. Six to 10 cross-sections were made by hand on the end of each stem segment and the remaining sample was ground and used for bacteria quantification by plate counting on triphenyltetrazolium chloride agar medium (Kelman, 1954) supplemented with gentamicin. Whole root systems were observed using an epifluorescence microscope with GFP filter (Nikon, Eclipse 80i) to locate the areas where the bacterium was present. Colonized roots were selected to be observed by confocal microscopy. Stem cross-sections and selected roots from each plant were placed on a glass slide, surrounded with solid vaseline and covered with agarose 1% used as immersion medium. Samples were observed using a confocal microscope (Leica, TCS SP5).

### Differential Staining of Stems Cross-Sections

#### Safranin-Fast Green Stain

Anatomical features of control and infected seedlings from the tolerant clone 09509.6 and the susceptible potato cultivar Chieftain were investigated by means of differential stains and analysis under a light microscope. Fresh plant material was fixed in FAA solution (50% ethanol, 5% glacial acetic acid, 30% formaldehyde and 15% water), dehydrated in an increasing ethanol and ethanol/xylene concentrations solutions and embedded in paraffin (Johansen, 1940). Cross sections of 10 μm were obtained with a Minot microtome. Sections were stained with safranin-fast green (Strittmatter, 1979), mounted in Canada Balsam Natural (Biopack) and observed using a light microscope (Axiolab, Zeiss MC 80).

#### Callose Detection

The detection of callose deposits was made harvesting plants 48 h after stem inoculation and cleaned during a whole night in 96% ethanol in Petri dishes. Once the stems were completely distained, they were cut manually and were incubated first in sodium phosphate buffer (0.07 M, pH 9) for 30 min, and then in aniline blue solution (0.05%) for 60 min (Daurelio et al., 2009). Finally, the samples were mounted in glycerol-water mix (50%) and observed immediately using an UV-fluorescence microscope (MIKOBA F320 with mercury lamp power box).

#### ROS Detection

Stem inoculation assays using UY031 strain were performed on the tolerant clone 09509.6 and susceptible cultivar Chieftain. Three clonal replications of each genotype were inoculated with the bacterial suspension, and negative plant controls were inoculated using sterile saline solution. Stems were harvested 24 h after inoculation and stained with DAB-HCl for 18 h in darkness. Then stems were placed in 96% ethanol to distain (Daurelio et al., 2009). Once the stems were completely distained, they were cut in cross sections. Reactive oxygen species (ROS) were detected using a light microscope (Zeiss MC 80, Axiolab).

### Data Analysis

Analysis of variance (ANOVA) and the Tukey’s multiple comparison test were applied with a 95% confidence level to analyze AUDPC, luminescence and plate counts values. Model residuals were used to check for the assumptions of normality and homogeneity of variances. Data from replicate trials of experiments were combined when there were no significant effects among trials. All statistical analyses were done using Infostat (Di Rienzo et al., 2009).

## Results

### Selected Potato Clones Were Classified As Susceptible or Tolerant Based on Wilting Scoring and Occurrence of Latent Infections

Experimental conditions used for resistance evaluation were favorable for distinguishing different levels of bacterial wilt resistance among the selected interspecific clones (**Table 1**). As expected, the potato cultivar Chieftain showed a highly susceptible response, with first symptoms appearing 5–7 days after inoculation and all plants completely wilted at the end of the experiment (data not shown). Based on comparison of AUDPC data, the clone 13001.79 was classified as susceptible, as only a low proportion (5–20%) of plants remained asymptomatic 28 days after inoculation in the repeated experiments. The other selected clones (13001.107, 11201.27, and 09509.6) showed significant differences in symptom progression compared to the susceptible control. For these genotypes asymptomatic plants predominated (>70%), and the pathogen was detected at the basal part of the stems revealing the occurrence of latent infections. Consequently, these clones were classified as tolerant.

**Table 1:** Bacterial wilt responses of selected potato genotypes expressed as the area under disease progress curve (AUDPC) and pathogen detection in stem tissues of asymptomatic plants 28 days post inoculation.

Genotype	Description^a^	AUDPC^b^	Stem latent infection^c^	Plant reaction
cv. Chieftain	tbr	62,6 A	-	Susceptible
13001.79	(cmm × cmm)	48,5 A	-	Susceptible
13001.107	(cmm × cmm)	17,1 B	+	Tolerant
11.201.27	(cmm × phu) × tbr	23,9 B	+	Tolerant
09.509.6	[(cmm × phu) × tbr] × tbr	15,8 B	+	Tolerant


^a^*Pedigree of each potato genotype. cmm: Solanum commersonii, phu: Solanum phureja, tbr: Solanum tuberosum*.

^b^*AUDPC values are means of two repeated experiments with two replicate trays containing eight plants of each genotype. Data were pooled across experiments since no significant effects involving trials were found in the analyses of variance (ANOVAs). Values followed by the same letter in the same column are not significantly different (Tukey’s multiple comparison test, P = 0.05)*.

^c^*Stem latent infection was evaluated in asymptomatic plants 28 days after inoculation with Ralstonia solanacearum. (+): growth of typical Ralstonia solanacearum colonies in mSMSA plates. (-): no evidence of Ralstonia solanacearum growth in mSMSA plates*.

### Tolerant Clones Showed a Restricted Colonization Pattern in Roots and Stem Base

**FIGURE 1:**
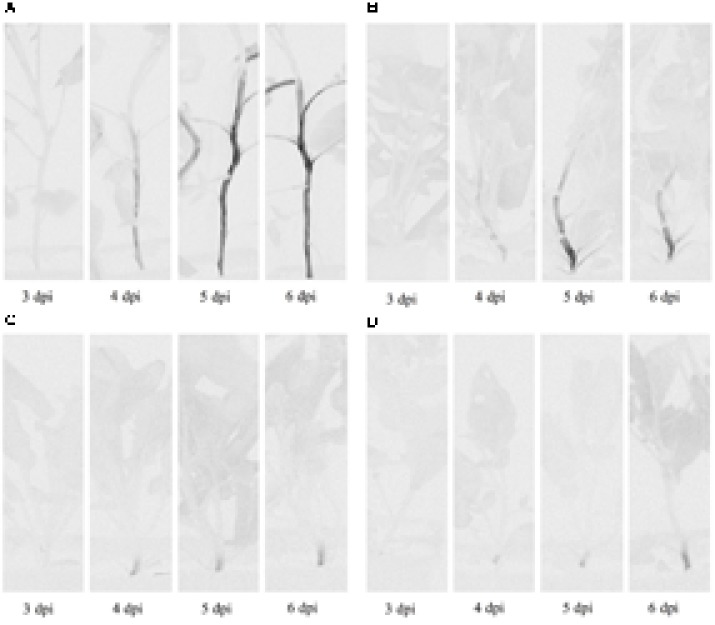
Bioluminescence imaging of *Ralstonia solanacearum* strain UY031 Pps-lux colonization pattern in different potato genotypes. **(A)** Susceptible potato cultivar *Solanum tuberosum* cv. Chieftain. **(B)** Susceptible potato clone 13001.79. **(C)** Tolerant potato clone 13001.107. **(D)** Tolerant potato clone 11201.27. Images were acquired 3, 4, 5, and 6 days post inoculation (dpi) using an *in vivo* imaging system. Light gray indicates background luminescence due to chlorophyll and black regions are tissue areas colonized by light-emitting bacteria.

**FIGURE 2:**
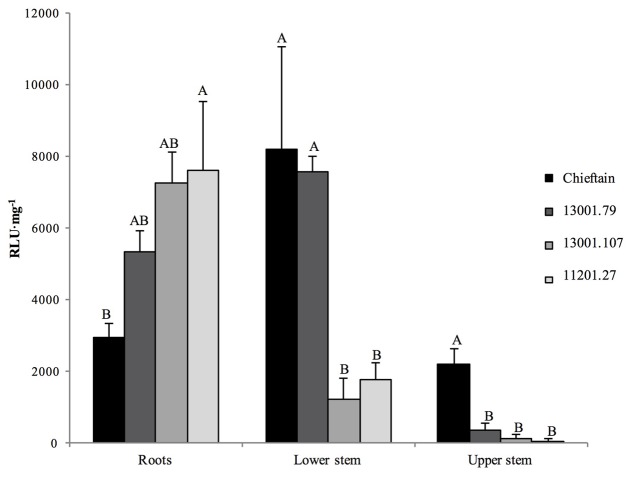
Bioluminescence quantification in roots and stems sections of potato plants 6 days after soil inoculation with *R. solanacearum* strain UY031 Pps-lux. Light emission is presented as relative luminescence units per milligram of plant fresh tissue (RLU⋅mg^-1^). Each column represents the mean luminescence (*n* = 6) detected in roots and stems sections of the susceptible potato cultivar *S.tuberosum* cv. Chieftain and interspecific potato breeding lines with different levels of bacterial wilt resistance including susceptible (13001.79) and tolerant (13001.107 and 11201.27) clones. Columns with the same letter within each sample type (roots, lower stem, upper stem) are not significantly different according the Tukey’s multiple comparison test (*P* = 0.05). Vertical bars represent standard errors of the means.

Plants of Chieftain and 13001.79 showed wilting symptoms 6 days after inoculation and the pathogen could be detected *in planta* as dark zones along the stem (**Figures 1A,B**). In contrast, plants of tolerant clones remained asymptomatic and bacterial colonization was observed only in the lower stem (collar) from day fourth after inoculation (**Figures 1C,D**). Luminescence emitted by UY031 Pps-lux strain 6 days after inoculation in infected plant tissues is shown as relative luminescence units (RLU) per milligram in **Figure 2**.

The tolerant clone 09509.6 displayed higher luminescence values in roots than the cultivar Chieftain (*P* = 0.0277), and clones 13001.79 and 13001.107 showed an intermediate response. In the lower stem (collar) both susceptible genotypes (13001.79 and Chieftain) showed higher bacterial loads than tolerant clones (13001.107 and 11201.27) (*P* = 0.0014). Luminescence was also measured in upper stems where the susceptible cultivar Chieftain showed the highest colonization level, while the other genotypes displayed luminescence values just above the background level.

### *Ralstonia solanacearum* Multiplied in a Limited Number of Xylem Vessels and Reached Low Population Densities in Stems of Tolerant Plants

**FIGURE 3:**
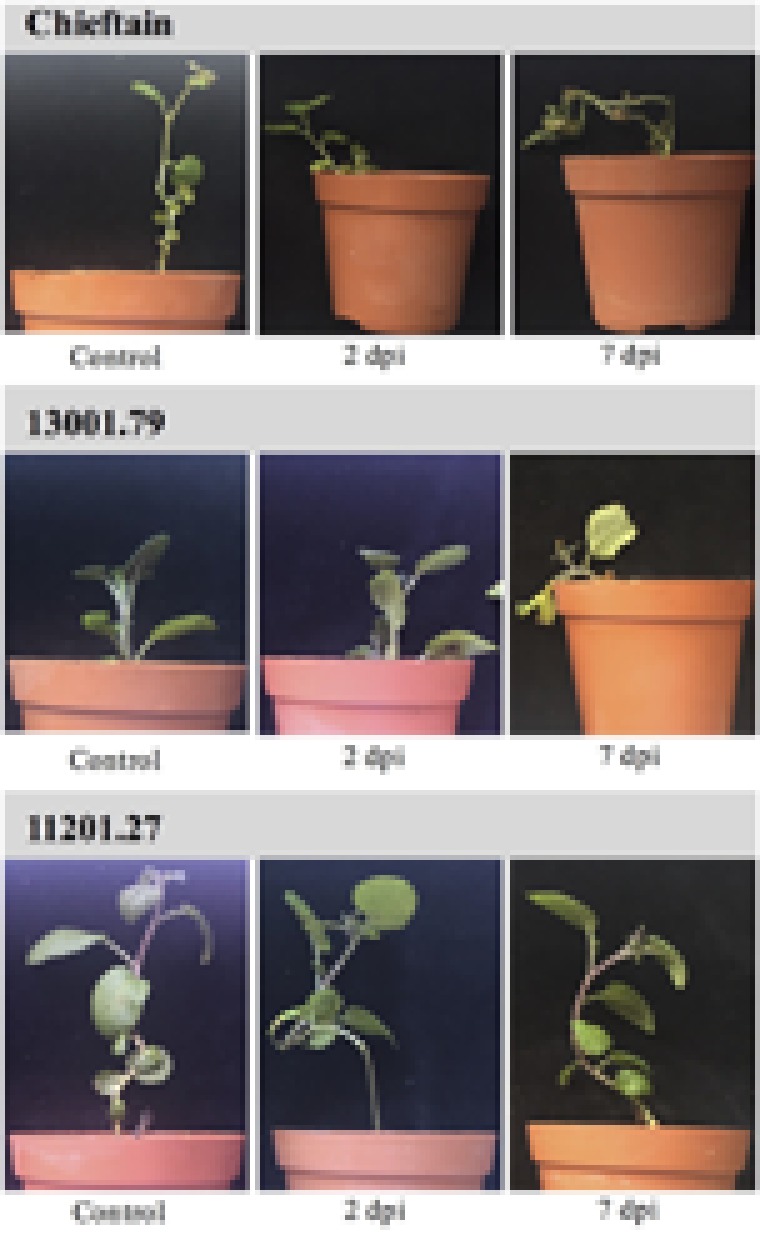
Symptom evaluation of bacterial wilt on potato plants soil inoculated with *R. solanacearum* strain UY031 Pps-GFP. Light pictures of plants from susceptible potato cultivar *S. tuberosum* cv. Chieftain, the susceptible clone 13001.79 and the tolerant clone 11201.27 were taken 2 and 7 days post inoculation (dpi). Control: mock inoculated plants of each genotype.

Two days after inoculation with the UY031 Pps-GFP reporter strain all plants remained asymptomatic (**Figure 3**), and the pathogen was not observed by microscopic evaluation neither in stems nor in roots. Five days later, wilting symptoms were evident only in susceptible plants (Chieftain and 13001.79) (**Figure 3**). At this time point bacterial colonization was verified in roots systems of all plants compared with mock inoculated roots of each variant. Representative images showed the same distribution pattern in roots of susceptible and tolerant clones (Supplementary Figure 1). A high frequency of wounded roots was observed highlighting the severity of the inoculation procedure.

**FIGURE 4:**
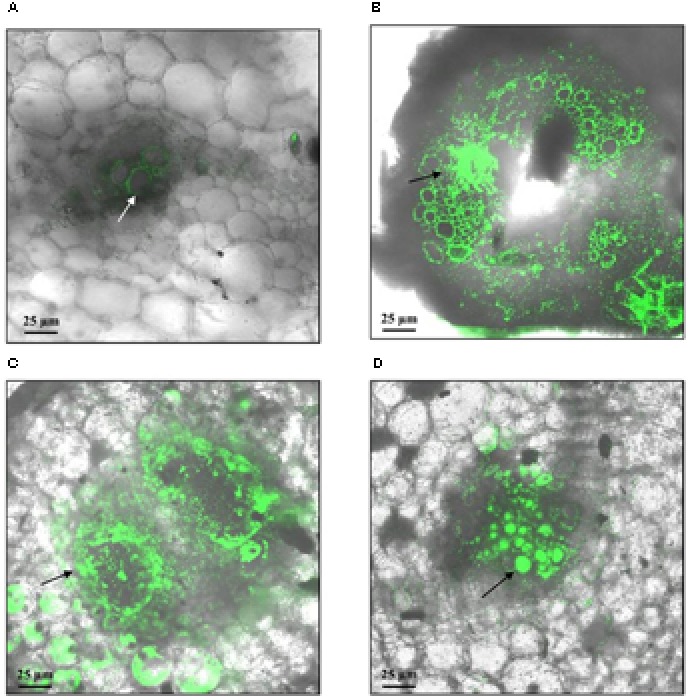
Representative confocal fluorescence micrographs of stem cross-sections of potato plants soil inoculated with *R. solanacearum* strain UY031 Pps-GFP. Bacterial colonization was evaluated 7 days after inoculation, in the susceptible potato cultivar *S. tuberosum* cv. Chieftain and interspecific potato breeding lines with different levels of bacterial wilt resistance including susceptible (13001.79) and tolerant (11201.27) clones. **(A)** Mock inoculated Chieftain plant. **(B)** Chieftain plant inoculated with *R. solanacearum*. **(C)** 13001.79 plant inoculated with *R. solanacearum*. **(D)** 11201.27 plant inoculated with *R. solanacearum*. Dark arrows show bacterial colonization and white arrow shows autofluorescence of xylem vessels.

On the contrary, differences in colonization patterns were observed among stems of susceptible and tolerant plants. In mock inoculated plants, stem sections were typically observed as few autofluorescent patches and representative xylem vessels identified by their roughly octagon shaped lignified cell walls (**Figure 4A**). Microscopic evaluation of susceptible plants with visible wilting symptoms showed a heavy colonization 7 days post inoculation. Bacteria was found in the vascular and parenchymatic tissues and distributed throughout the apoplast (**Figures 4B,C**). In contrast, representative images of asymptomatic plants from the tolerant clone 11201.27, showed bacterial cells occluding a limited number of xylem vessels within only one of the vascular bundles (**Figure 4D**). This restricted distribution may be associated with a reduced interference of water transport explaining the typical absence of wilting symptoms in this clone. For other tolerant clones (13001.107 and 09509.6) all plants remained asymptomatic and no bacterial cells were observed in the transverse sections of stems by confocal microscopy (data not shown).

**FIGURE 5:**
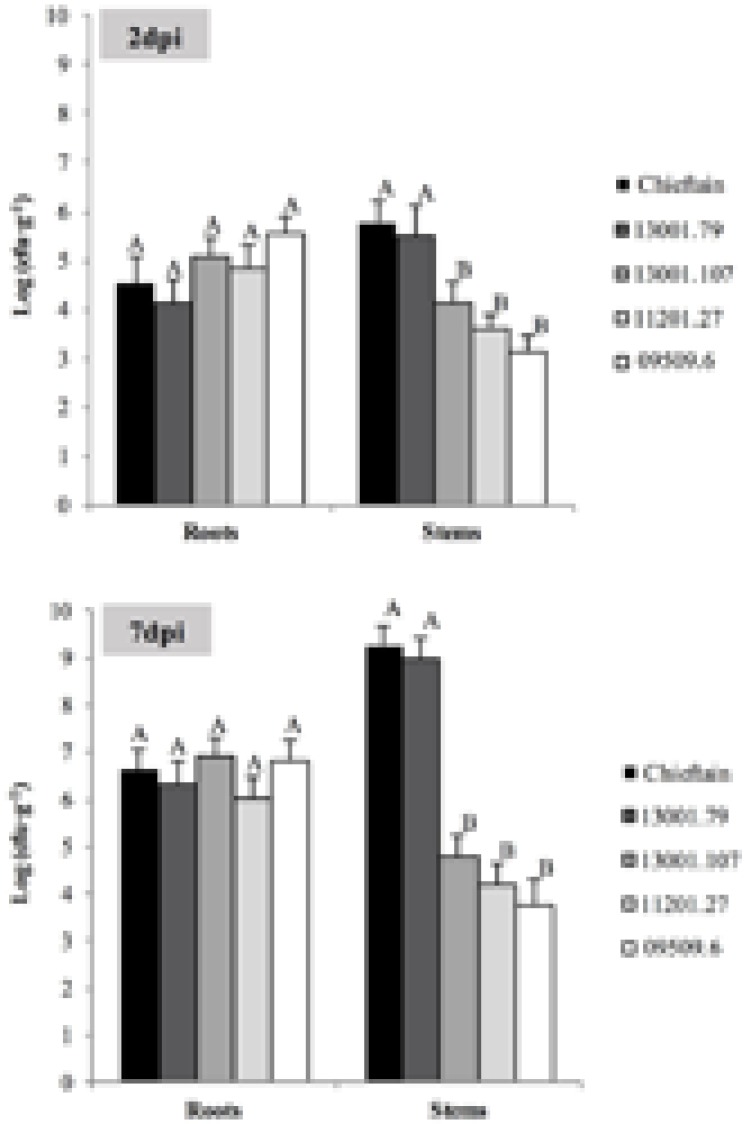
Bacterial populations in roots and stems of susceptible and tolerant potato genotypes 2 (A) and 7 (B) days post inoculation (dpi) with *R. solanacearum* strain UY031 Pps-GFP. Each column represents the mean bacterial load (*n* = 6) determined by plate counting in roots and stems samples of the susceptible potato cultivar *S. tuberosum* cv. Chieftain and interspecific potato breeding lines with different levels of bacterial wilt resistance including susceptible (13001.79) and tolerant (13001.107, 11201.27, and 09509.6) clones. Columns with the same letter within each sample type (roots, stems) are not significantly different according the Tukey’s multiple comparison test (*P* = 0.05). Vertical bars represent standard errors of the means.

Microscopy provides valuable qualitative observations but is not sensitive enough and does not allow quantification. Hence, the same roots and stems samples were also used for quantitative analysis of pathogen colonization by plate counting. Two days after inoculation roots from all clones were already colonized by the pathogen, although microscopic evaluations failed to detect *R. solanacearum* cells in plant tissues (**Figure 5**). No significant differences were observed among pathogen densities in roots of all tested clones 2 and 7 days post inoculation. However, susceptible plants showed higher bacterial loads in stems compared to plants from the tolerant clones. In plants from the susceptible cultivar Chieftain and clone 13001.79, *R. solanacearum* multiplied extensively in the stems, and quickly increased to 10^6^ cfu⋅g^-1^ 2 days after inoculation and reached more than 10^9^ cfu⋅g^-1^ 7 days after inoculation. In tolerant clones (13001.107, 11201.27, and 09509.6), there was no apparent increase in *R. solanacearum* population in stems from 2 to 7 days after inoculation. This is consistent with the fact that that no wilting symptoms were observed in these plants throughout the study. In these clones, bacterial titers in stems tissues reached an average of 10^4^ cfu⋅g^-1^, which is probably below the required levels for disease development.

### Histological Effects of *R. solanacearum* Infection in Susceptible and Tolerant Potato Plants

**FIGURE 6:**
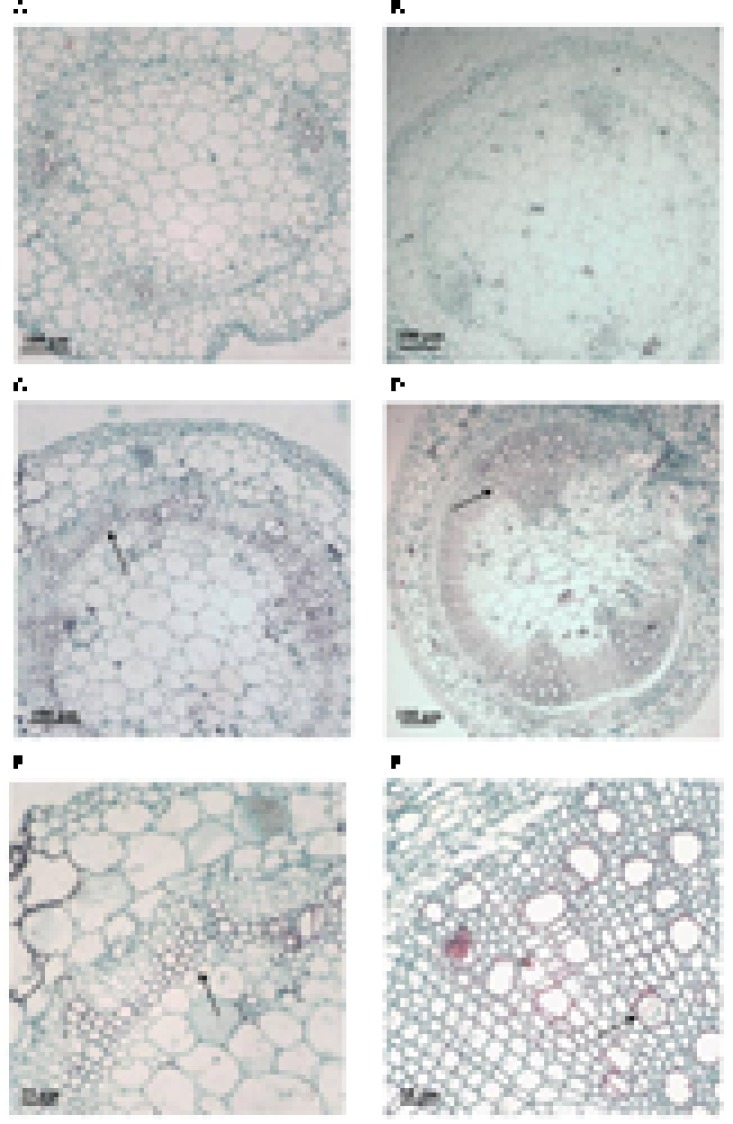
Representative light micrographs of safranin-fast green stained stem cross-sections of plants from the susceptible potato cultivar *S. tuberosum* cv. Chieftain and the tolerant clone 09509.6. Xylem vessels architecture was evaluated 5 days after soil inoculation with *R. solanacearum* UY031 strain and compared to mock inoculated plants. Safranin dyes secondary cell walls red and fast green dyes cellulose light blue. **(A)** Mock inoculated Chieftain plant. **(B)** Mock inoculated 09509.6 plant. **(C,E)** Chieftain plant inoculated with *R. solanacearum*. **(D,F)** 09509.6 plant inoculated with *R. solanacearum*. Dark arrows show hyperplasic activity and lignin deposition **(C–E)**, or xylem vessels occluded by tyloses with globular shape **(F)**.

Cell division with the generation of increased quantity of conductor tissue (xylem and phloem) was observed strongly in infected plants of the tolerant clone 09509.6 (**Figure 6D**). The susceptible cultivar Chieftain also showed hyperplasic activity after *R. solanacearum* infection but to a lesser extent (**Figure 6C**). Mock inoculated controls are shown in **Figures 6A,B**. In addition, stems from clone 09509.6 presented the highest level of lignification, with an increased thickening of xylem vessels compared to the susceptible cultivar Chieftain. This was revealed by staining with safranin which dyes secondary cell walls red (**Figures 6E,F**). Representative images of thin sections of infected stems from clone 09509.6 also revealed the existence of vessels plugged by tyloses with a globular shape (**Figure 6F**). This result suggests that infected xylem vessels could induce these structures to occlude the vascular system in tolerant plants limiting bacterial flow to upper tissues. In mock inoculated or susceptible plants no tylose production was observed.

**FIGURE 7:**
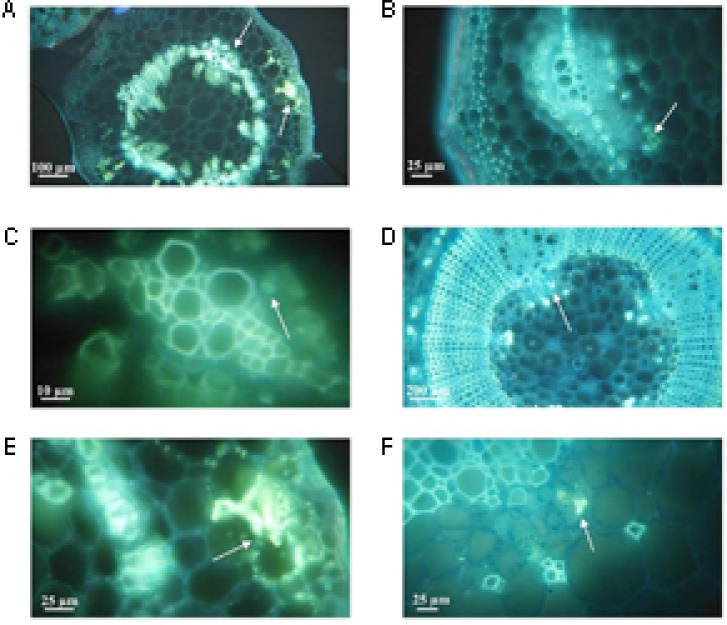
Representative fluorescense (GFP) micrographs of aniline blue stained stem cross-sections of plants from the susceptible potato cultivar *S. tuberosum* cv. Chieftain and the tolerant clone 09509.6. Callose deposition was detected as yellow fluorescence zones (white arrows) 2 days after stem inoculation with *R. solanacearum* UY031 strain and compared to mock inoculated plants. **(A)** Chieftain plant inoculated with *R. solanacearum.* **(B)** Mock inoculated 09509.6 plant. **(C)** Xylem vessels of Chieftain plant inoculated with *R. solanacearum.* **(D)** 09509.6 plant inoculated with *R. solanacearum*. **(E)** Cortical parenchyma of Chieftain plant inoculated with *R. solanacearum.* **(F)** Internal phloem tissue of tolerant potato genotype 09509.6 inoculated with *R. solanacearum.*

Callose was localized using aniline blue solution leading to yellow fluorescence (**Figure 7**). In infected plants from the susceptible cultivar Chieftain pads of callose were observed filling the sieve tubes in phloem tissue and in areas of cellular communication between cortical parenchyma cells (**Figures 7A,C,E**). Callose deposits were not observed in mock inoculated plants (data not shown). In the tolerant clone 09509.6 callose was abundant in both healthy and infected plants, and was located filling the sieve tubes in the phloem tissue (**Figures 7B,D,F**). In infected plants of this clone the increased quantity of conductor tissue due to induced hyperplasic activity was revealed as strong autofluorescence of the lignified tissue (**Figure 7D**).

**FIGURE 8:**
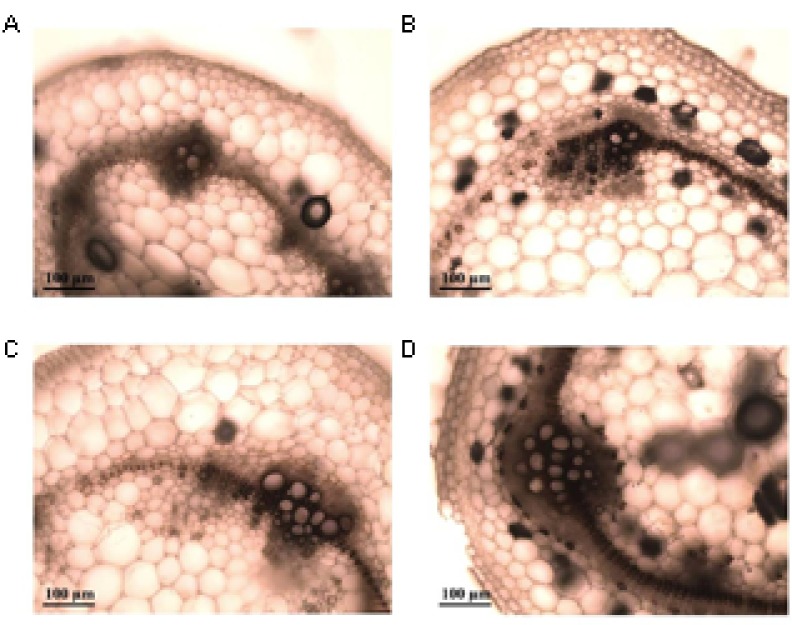
Representative light micrographs of diamino benzidine (DAB) stained stem cross-sections of plants from the susceptible potato cultivar *S. tuberosum* cv. Chieftain and the tolerant clone 09509.6. Reactive oxygen species (ROS) production was evaluated 24 h after stem inoculation with *R. solanacearum* UY031 strain and compared to mock inoculated plants. DAB forms a brown precipitate in presence of hydrogen peroxide, indicating ROS production. **(A)** Mock inoculated Chieftain plant. **(B)** Mock inoculated 09509.6 plant. **(C)** Chieftain plant inoculated with *R. solanacearum*. **(D)** 09509.6 plant inoculated with *R. solanacearum*.

Diamino benzidine (DAB) formed a brown precipitate with hydrogen peroxide that was correlated with production of ROS (**Figure 8**). In susceptible cultivar Chieftain no differences were observed between healthy and infected plants (**Figures 8A,C**). The tolerant clone showed a stronger and more extended ROS production after pathogen infection compared to the mock inoculated plants (**Figures 8B,D**). In infected plants of this clone the brown precipitate revealing ROS production was mainly observed around the conductor tissue and throughout the apoplast (**Figure 8D**).

## Discussion

Breeding programs focused on the development of bacterial wilt resistant potato varieties are hampered by the scarcity of stable resistance sources against *R. solanacearum.* In this study we present the evaluation of selected interspecific clones from the potato breeding program developed in Uruguay, based on the introgression of resistance from *S. commersonii.* This wild species was previously reported to carry resistance against *R. solanacearum* (Laferriere et al., 1999; Carputo et al., 2009), however, these studies were limited to only one or few accessions, and the resistance sources were not further improved considering required agronomic and commercial traits. In contrast, our national potato breeding program makes use of the high genetic diversity available in this species which is widely distributed and adapted to our environmental conditions (Pianzzola et al., 2005; Siri et al., 2009, 2011; Gaiero et al., 2017).

The challenge of advanced pre-breeding materials belonging to different backcross populations showed consistent results in repeated experiments, attesting to the reliability of the wounded-roots soil inoculation procedure and the contrasting responses against *R. solanacearum* infection. Based on wilting scoring and detection of latent infections, the evaluated clones were classified as susceptible (similar disease progression than a susceptible potato cultivar) or tolerant (when most plants replicates remain asymptomatic 28 days post inoculation and the pathogen is present in stems). Interestingly, some breeding lines even after one or two backcrosses with susceptible *S. tuberosum* germplasm presented low level of bacterial wilt incidence and therefore maintained the resistance from the wild species.

Asymptomatic latent infections caused by *R. solanacearum* should be considered in potato breeding programs to avoid the selection of tolerant varieties which would promote pathogen dissemination under favorable environmental conditions (Priou et al., 2005). This problem is not exclusive for potato and was observed in previous studies for other hosts of *R. solanacearum* including pepper, tomato, eggplant, and geranium (Swanson et al., 2005; Lebeau et al., 2011; Heshan et al., 2017). By assessing phenotypes based on wilting symptoms and pathogen detection in plant tissues, it is possible to differentiate two mechanisms of defense: plant resistance based on limitation of pathogen access to the vascular system (immunity) and resistance based on plant survival harboring the bacteria within xylem vessels (latent infection or tolerance) (Lebeau et al., 2011). Resistance screening of potato germplasm derived from the wild species *S. commersonii* did not reveal immunity to *R. solanacearum.* However, it is important to consider that the assay used in this study for resistance evaluation is more severe than usual field conditions, as the plantlets have thinner stems and limited rooting systems, the pathogen is present at high concentration in soil (10^6^ cfu⋅g^-1^), the roots are artificially damaged and the incubation conditions are optimal for disease development.

The occurrence of asymptomatic infections in susceptible potato cultivars may be a way of pathogen dissemination, particularly in temperate growing regions with slower disease progress. Introducing the evaluation of latency in our breeding program aimed to avoid a selection only based on wilting symptoms. In addition, pollen fertility, tuber quality and other agronomic traits are also being considered when selecting the best parental material for future crosses. Although no truly resistant genotypes were found in this study, partial resistant or highly tolerant clones showing a low proportion of wilted plants and restricted pathogen colonization should be considered as a valuable genetic resource for breeding. The usefulness of these clones would be appreciated in severely infected tropical lands, where acceptable potato yields would only be achieved with them. This extent and the use of the harvested tubers as potato seeds should be further studied.

Methods allowing localization and visualization of microbes have account for a substantial progress in the understanding of the interactions between pathogen and its host plants. The ability of *R. solanacearum* reporter strain UY031 Pps-lux to emit bioluminescence *in planta* reported by Cruz et al. (2014), was here extended with the implementation of a fluorescent GFP-tagged *R. solanacearum* strain (UY031 Pps-GFP) as an additional tool for pathogen localization within infected tissues. To achieve strong and stable expression of the reporter systems, *gfp* and *lux* genes were integrated in a neutral position of the *R. solanacearum* genome under the control of a constitutive plastid promotor (*psbA*) (Wang et al., 2007; Monteiro et al., 2012). GFP-labeled reporter strains have distinct advantages, including the ability to detect bacteria at the single-cell level when are used in combination with microscopic observations (Kohlmeier et al., 2007). On the other hand, luminescent reporters are more sensitive, allows for a non-destructive *in vivo* imaging, and quantification of the emitted luminescence could be correlated with bacterial loads in infected tissues (Cruz et al., 2014).

Both reporter strains were readily detected in potato plants with visible wilting symptoms. In susceptible clones the transition into a symptomatic stage relied on extensive pathogen multiplication both in lower and upper stem segments, reaching a high population density soon after inoculation. This situation was correlated with the observation of dense bacterial cells aggregates in stem parenchymatic tissues and filling a large proportion of xylem vessels causing a progressively lower water conduction ability. In our previous study using the UY031 Pps-lux reporter strain, tolerant *S. commersonii* plants remained asymptomatic after inoculation and showed high bacterial colonization in root systems but not in the stems (Cruz et al., 2014). In the present study, using the same reporter strain and inoculation procedures, luminescence was detected not only in the root systems but also in the stem base of asymptomatic plants. Since tolerant clones currently evaluated were obtained from backcross populations with the susceptible parent *S. tuberosum*, this extended pathogen distribution may be attributed to the differential genetic background compared to the *S. commersonii* accessions previously evaluated.

Results obtained in this study suggest that resistance in potato is clearly related with the host capability to restrict bacterial colonization and multiplication, particularly limiting dissemination along the stem. This is in agreement with previous observations in tomato resistant and tolerant genotypes (Grimault et al., 1994a; Nakaho et al., 2000, 2004). However, in these latently infected tomato plants, pathogen densities in stems were higher (10^5^–10^8^ cfu⋅g^-1^) (Grimault et al., 1994a), compared to bacterial loads reached in tolerant potato plants (10^3^–10^4^ cfu⋅g^-1^). It is probably that pathogen translocation from root to stem tissues and/or pathogen multiplication in stems are prevented in a more efficiently way in potato genotypes, leading to higher levels of resistance to bacterial wilt. Another difference between bacterial wilt resistance mechanisms in both crops refers to root colonization. It was recently reported that resistance in tomato is partly due to the ability of tolerant plants to restrict bacterial root colonization in space and time (Caldwell et al., 2017). However, results obtained in this study consistently showed no differences regarding the colonization and distribution pattern in root systems of susceptible and tolerant clones. This finding was obtained by luminescence quantification, confocal microscopy observations and plate counting, strongly suggesting that at least in these potato clones, limitation of pathogen infection occurs later on.

Plant resistance to pathogens is the consequence of interconnected constitutive and inducible defense responses. The possible infection paths and the molecular mechanisms underlying plant defenses have been recently reviewed for several xylem-colonizing pathogens, including *R. solanacearum* (Yadeta and Thomma, 2013; Bae et al., 2015). Plant cell wall is one of the first structural barriers that pathogens have to cross to successfully infect plant tissues (Miedes et al., 2014). Pathogens also need to breakdown cell walls as a source of nutrients for their growth once inside the host. Plants have evolved specialized mechanisms for detecting intruders and sensing the cell wall integrity. Pathogen recognition induces the cell wall remodeling to restrict pathogen colonization and spreading needed for disease control (Bellincampi et al., 2014). This process involves structural and chemical changes, including lignification, callose deposition, cell wall protein cross-linking, production of reactive oxygen species and antimicrobial compounds.

In tomato cultivars with resistance to bacterial wilt, physical barriers are involved in limitation of pathogen spread. In the resistant cultivar Caraıbo, many tyloses were found occluding pathogen-colonized and contiguous xylem vessels (Grimault et al., 1994b). On the other hand, tylose formation was not induced in infected tomato plants other resistant cultivar (L S-89) (Nakaho, 1997). In this cultivar, prevention of pathogen spread in plant tissues was related with the reinforcement of cell walls and the pit membranes, and also with the accumulation of electron-dense materials in vessels and around parenchyma cells (Nakaho et al., 2000).

Herein, the tolerant potato clone displayed significant structural responses after soil inoculation with *R. solanacearum.* Cell division with the generation of increased quantity of conductor tissue, lignin deposition and thickening of xylem vessels were clearly observed. In addition, in infected plants of this clone several vessels plugged by tyloses with globular shape were observed. This type of tyloses results from expansion of parenchyma cells associated to xylem vessels, probably preventing pathogen transportation within xylem vessels (Kpémoua et al., 1996).

Callose deposition and ROS production are additional first line responses in plant defense (Stone and Clarke, 1992; Lamb and Dixon, 1997). Callose is an amorphous polymer where antimicrobial compounds are depositated, leading to delivery of chemical defenses in specific points of attack (Luna et al., 2011). Although callose deposition contributes to plant immunity against many plant pathogens, it was reported that these structures were also found in sites of pathogen entry (Aist, 1976; Voigt, 2014). In this study, no differences were found between healthy and infected plants of the tolerant genotype, and callose was abundant even in mock inoculated plants. This finding suggests that constitutive callose deposition in these tolerant plants could contribute to reinforce the strength of plant cell walls preventing pathogen spreading.

Reactive oxygen species production is induced after several forms of biotic and abiotic stress. It has been suggested to prevent disease progress, either by directly causing pathogen death, or by promotion of a reinforcement of the cell wall through cross-linking of proteins and phenolics (Thordal-Christensen et al., 1997; Brown et al., 1998). In tomato, increased level of ROS production and lignin deposition in cell wall could promote bacterial wilt resistance in tomato (Mandal et al., 2011). Our results showed an induced ROS production after *R. solanacearum* infection in tolerant plants. However, quantitative evaluation of ROS production over time including additional potato genotypes is needed to further determine the implications of this plant defense response.

This study proved that tolerant potato clones may show none or few symptoms while being partially to highly colonized by *R. solanacearum* in roots and stems. Our results suggest that the restricted pathogen multiplication in stems of tolerant genotypes is a consequence of constitutive or induced structural and biochemical plant defense responses. However, several aspects of this plant-pathogen interaction, and the consequences of latent infection in potato resistance should be further investigated. This study highlights the potential of the selected tolerant potato interspecific clones as valuable genetic resources for potato-breeding programs and leads to a better understanding of resistance against *R. solanacearum* in potato.

## Author Contributions

VF, MP, FV, GG, MV, and MS conceived and designed the work. VF performed all the experiments, MT and MR contribute to histological evaluations. MP, FV, EO, MV, and MS provided reagents and materials. All authors contributed to analysis and interpretation of results. VF, MR, EO, GG, MV, and MS wrote the manuscript. All authors have made substantial, direct and intellectual contribution to the work, and approved it for publication.

## Conflict of Interest Statement

The authors declare that the research was conducted in the absence of any commercial or financial relationships that could be construed as a potential conflict of interest.
