# Supplementary material for: Interspecific Potato Breeding Lines Display Differential Colonization Patterns and Induced Defense Responses after Ralstonia solanacearum Infection
Source: Front Plant Sci. 2017 Aug 28;8:1424. doi: 10.3389/fpls.2017.01424 (PMC5581342; doi:10.3389/fpls.2017.01424)
Supplement: Supplementary file 1 [file Image_1.PDF]

Supplementary Material

Interspecific potato breeding lines display differential colonization patterns and induced defense responses after *Ralstonia solanacearum* infection

Virginia Ferreira[1], María Julia Pianzzola[1], Francisco Vilaró[2], Guillermo A. Galván[3], María Laura Tondo[4], María Victoria Rodriguez[5], Elena G. Orellano[4], Marc Valls[6,7], María Inés Siri[1*]

[1] Departamento de Biociencias, Facultad de Química, Universidad de la República, Montevideo, Uruguay.

[2] Unidad de Horticultura, Instituto Nacional de Investigaciones Agropecuarias, INIA Las Brujas, Canelones, Uruguay.

[3] Departamento de Producción Vegetal, Centro Regional Sur (CRS), Facultad de Agronomía, Universidad de la República, Canelones, Uruguay.

[4] Instituto de Biología Molecular y Celular de Rosario-CONICET, Rosario, Argentina. Área Biología Molecular, Facultad de Ciencias Bioquímicas y Farmacéuticas, Universidad Nacional de Rosario, Rosario, Argentina.

[5] Área Biología Vegetal-CONICET, Facultad de Ciencias Bioquímicas y Farmacéuticas, Universidad Nacional de Rosario, Rosario, Argentina.

[6] Center for Research in Agricultural Genomics, CSIC- IRTA- UAB -UB, Barcelona, Catalonia, Spain.

[7] Department of Genetics, Universitat de Barcelona, Barcelona, Catalonia, Spain.

*** Correspondence:**
María Inés Siri

msiri@fq.edu.uy

1 Supplementary Figures and Tables

1.1 Supplementary Figures

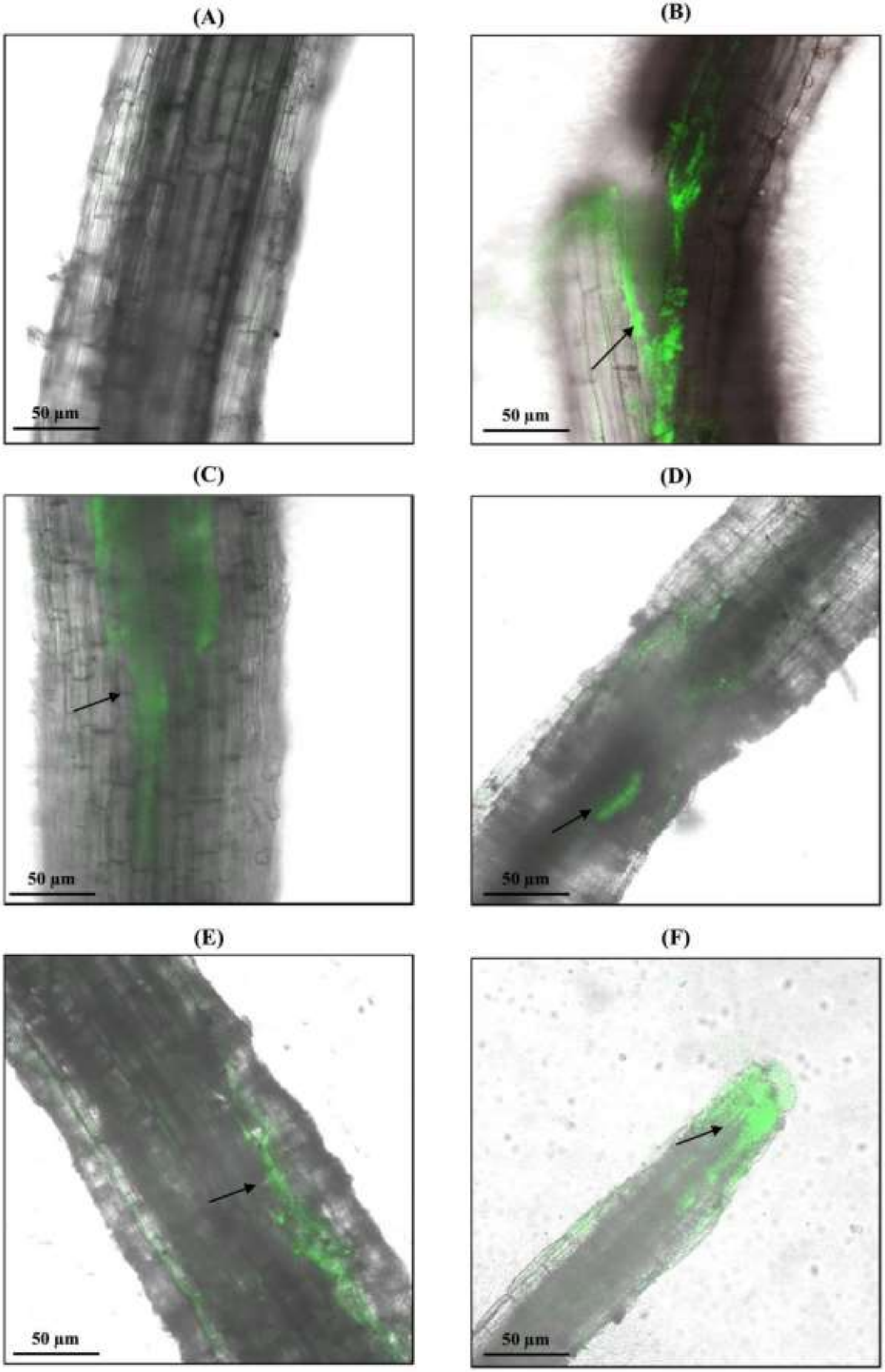
(A)
50 µm
(B)
50 µm
(C)
50 µm
(D)
50 µm
(E)
50 µm
(F)
50 µm

Supplementary Figure 1. Representative confocal fluorescence micrographs of roots of potato genotypes soil inoculated with *Ralstonia solanacearum* strain UY031 Pps-GFP. Bacterial colonization was evaluated seven days after inoculation, in the susceptible potato cultivar *Solanum tuberosum* cv. Chieftain and interspecific potato breeding lines with different levels of bacterial wilt resistance including susceptible (13001.79) and tolerant (13001.107, 11201.27, 09509.6) clones. (A): Mock inoculated Chieftain plant. (B): Chieftain plant inoculated with *R. solanacearum*. (C): 13001.107 plant inoculated with *R. solanacearum*. (D): 13001.79 plant inoculated with *R. solanacearum*. (E): 09509.6 plant inoculated with *R. solanacearum*. (F): 11201.27 plant inoculated with *R. solanacearum*. Dark arrows show bacterial colonization.
